# Sick leave after transient ischemic attack among working-age adults in Sweden: A register-based cohort study

**DOI:** 10.1371/journal.pone.0359635

**Published:** 2026-09-30

**Authors:** Adam Viktorisson, Malin Reinholdsson, Maja Bengtsson, Tamar Abzhandadze, Katharina S. Sunnerhagen

**Affiliations:** 1 Institute of Neuroscience and Physiology, Rehabilitation Medicine, University of Gothenburg, Sweden; 2 Department of Rehabilitation Medicine, Sahlgrenska University Hospital, Gothenburg, Sweden; 3 Department of Occupational Therapy and Physiotherapy, Sahlgrenska University Hospital, Gothenburg, Sweden; Duke University Medical Center: Duke University Hospital, UNITED STATES OF AMERICA

## Abstract

**Background:**

A transient ischemic attack (TIA) is a brief episode of neurological dysfunction caused by focal brain or retinal ischemia, with symptoms resolving within 24 hours and without evidence of acute infarction. This study aims to investigate patterns of sick leave before and after TIA in working-age adults compared with matched general population controls.

**Methods:**

This register-based cohort study included all individuals aged ≤66 years treated for TIA in Gothenburg, Sweden, between 2014 and 2019, and age- and sex-matched general population controls (4:1). Data from national registers were used to assess sick leave the year before and after the index event. Logistic regression was used to model associations of sick leave after TIA.

**Results:**

347 individuals with TIA (median age 59 years; 40.6% women) and 1,388 controls were included. Individuals with TIA had more days on sick leave than controls during the year before the event (mean 26 [SD 78] vs. 14 [SD 55], p < 0.001), and increased by 39 days during the year after TIA compared with the year before (95% CI 28–50 days, p < 0.001). The number of individuals on sick leave peaked 14 days after TIA (35.7% vs. 3.4% controls) and remained elevated at 1 year (11.5% vs. 3.8%). The most common registered sick-leave diagnoses 14 days after the event were TIA (43%), stroke (12%), and mental health conditions (13%). Higher income (middle tertile: adjusted OR 2.54, 95% CI 1.22–5.29; highest: 2.11, 1.03–4.34) and employment (adjusted OR 3.11, 1.23–7.88) were associated with sick leave at 14 days.

**Conclusions:**

Over one-third of working-age adults were on sick leave 2 weeks after TIA, and one in ten remained on benefit after one year. Individuals with TIA also tend to have more days on sick leave the year before TIA compared to the general population.

## Introduction

Transient ischemic attack (TIA) is commonly defined as a brief episode of neurological dysfunction caused by focal brain or retinal ischemia, with clinical symptoms resolving within 24 hours, typically lasting less than one hour, and without evidence of acute infarction [1]. Consequently, patients with TIA should have full recovery without lasting impairment. Growing evidence, however, suggests that many patients diagnosed with these criteria experience residual cognitive, psychological, or physical difficulties [2–5]. Residual consequences after TIA may also affect daily functioning and the ability to return to work, whether fully, partially, or with modifications [6].

Research on occupational outcomes and sick leave following TIA remains limited, although a prior study found that about one in five of working-aged individuals with TIA have ongoing sick leave 5–12 weeks following the event [7]. Comprehensive data and comparisons with population-based controls are lacking. Understanding patterns of sick leave before and after TIA may reveal hidden morbidity and expose rehabilitation needs in this population.

The aim of this study was to investigate patterns of sick leave before and after TIA in working-age adults compared with matched general population controls, and to identify determinants of sick leave after TIA.

## Methods

This longitudinal, register-based cohort study included all patients aged ≤66 years treated for TIA at the three stroke units of Sahlgrenska University Hospital in Gothenburg, Sweden, between 1 November 2014 and 31 December 2019. The upper age limit of 66 years was chosen to reflect working age in Sweden during the study (the statutory old-age retirement age). All patients with TIA were identified through the regional Väststroke quality register with a catchment area of approximately 800,000 inhabitants. A matched control population was drawn from the Total Population register held by Statistics Sweden. Control individuals were matched 4:1 to TIA cases by exact age and sex. Each control was assigned the same index date as their corresponding TIA case, defined as the date of hospitalization for the case.

Additional data were obtained from the national Swedish stroke register (Riksstroke) [8], the Longitudinal Integration Database for Health Insurance and Labour Market Studies (LISA) [9], and the Swedish Social Insurance Agency, which provided information on sick leave (Swedish terminology: sickness benefit) and disability pension (Swedish terminology: sickness compensation). All data were pseudonymized before being delivered to the researchers on April 13, 2023.

Ethical approval was granted by the Swedish Ethical Review Authority (2021−03324, approved July 7, 2021; and amendments 2022-02747-02, approved May 21, 2022 / 2022-06041-02, approved November 27, 2022). In Sweden, data collection in national quality registries is permitted without individual informed consent under the Personal Data Act (SFS 1998:204). Data cannot be shared publicly because of Swedish regulations. Requests for access this data may access may be submitted to the Sahlgrenska Academy, at University of Gothenburg: info@sahlgrenska.gu.se. Access is subject to independent review and approval by the relevant data holders and the Swedish Ethical Review Authority, as applicable. The study data and documentation are stored in a secure University of Gothenburg research environment and retained in accordance with institutional archiving requirements.

### Sick leave and disability pension

In Sweden, employers provide sick pay during the first 14 days of a sick-leave period. Thereafter, financial compensation for sick leave is administered by the Swedish Social Insurance Agency, a national public authority. During the return-to-work process, the agency applies a stepwise rehabilitation model to assess work capacity, with increasingly stringent criteria applied over time. Participants were classified as being on sick leave if they had an active benefit episode at any of the following time points: 30 days before the index date, at the index date, or at 14 days, 2 months, or 1 year after the index date. All degrees of sick leave (25%, 50%, 75%, or 100%) were included in the definition of receiving the benefit. For each sick leave episode, diagnostic codes (ICD-10) reported by the certifying physician were available and used to identify the medical cause of the absence. For participants on sick leave, ICD-10 codes were categorized into the following groups: TIA (G45), Stroke (I61, I63, I64), Cardiovascular (other I codes), Neurological (other G codes), Musculoskeletal (M), Respiratory (J), Mental health (F), and Cancer (C). Codes that did not fit any of these predefined categories were labeled as Other, while sick leave without any registered ICD-10 code were classified as Unknown.

Disability pension (previously commonly referred to as early retirement) may be granted instead of sick leave when return to work is considered unlikely due to long-term illness or disability. However, returning to work always remains a possibility. The presence of disability pension was recorded both before and during the year after the index date.

### Employment status

Employment status was derived from the LISA and reflects the official classification of employment in November each year, based on the Swedish Register-based Labour Market Statistics (RAMS), which approximates the International Labour Organization definition of employment (having performed income-generating work for at least one hour during a reference week). The value from the calendar year preceding the index date was used, ensuring that employment status was measured before the index event. Individuals classified as employed or non-employed. Eligibility for paid sick leave in Sweden is generally based on the presence of sickness-benefit generating income (SGI), which applies to all individuals applying for sick leave regardless of employment status; thus, both employed and unemployed individuals may be denied compensation in the absence of SGI. Unemployed individuals may receive compensation during their sick leave if they have an established SGI and are actively registered as job seekers with the Public Employment Service.

### Income and education

Income was defined as disposable income per individual, obtained from LISA. Disposable income represents the sum of all taxable and tax-free income, including wages, business income, capital income, and social transfers, minus taxes and negative transfers (e.g., repaid study loans or paid maintenance support). In the total study population (individuals with TIA and controls), yearly disposable income was divided into tertiles. The lowest tertile corresponded to <222,900 SEK and the highest tertile to >330,800 SEK. Educational level was also collected from LISA and categorized according to the highest completed level: < 10 years (primary education), 10–12 years (upper secondary education), and >12 years (post-secondary or higher education).

### TIA specific variables

In the TIA cohort medical conditions, including prior stroke, atrial fibrillation, hypertension, and diabetes, were obtained from the Riksstroke register. Information on smoking status was also collected from Riksstroke. Data on prior physical activity were retrieved from the regional Väststroke register and were based on a self-reported assessment scale conducted by physiotherapists at the stroke unit. Physical activity was defined as light activity ≥4 hours per week or moderate activity ≥2 hours per week during the year preceding the event, according to the Saltin–Grimby Physical Activity Level Scale [10].

### Statistics

Baseline characteristics of individuals with TIA and matched controls were summarized using median and ranges for continuous variables and counts with percentages for categorical variables. The prevalence of sick leave and disability pension at five predefined time points (30 days before the index date, at the index date, 14 days, 2 months, and 1 year after the index date) was calculated separately for TIA cases and controls.

The number of days on sick leave during the year preceding the index date was compared between individuals with TIA and controls using the Wilcoxon rank-sum test. The Spearman rank correlation was used to examine the association between the number of sick leave days before and after TIA. Differences in sick leave days during the year before and after TIA was examined using linear regression model, estimating the mean within-person change and corresponding 95% confidence interval.

Associations between individual characteristics and being on sick leave 14 days after a TIA were first examined using univariate binary logistic regression. Individuals with prior disability pension and cases with missing data in any of the included variables were excluded. Variables with a univariate p-value <0.2 in addition to age and sex were considered for inclusion in a multivariable model. Multicollinearity was assessed using the variance inflation factor (VIF), with no evidence of problematic collinearity (all VIF < 3). Model fit was evaluated using Nagelkerke’s R^2^ and the Hosmer–Lemeshow goodness-of-fit test, which indicated acceptable model calibration. Residual and influence diagnostics did not reveal any violations of model assumptions.

All statistical analyses were performed using R version 4.4.1 (R Foundation for Statistical Computing, Vienna, Austria), and statistical significance was defined as p < 0.05 (two-sided).

## Results

A total of 347 individuals aged ≤66 years with TIA were included, together with 1,388 age- and sex-matched controls (median age 59 years, range 26–66; 40.6% women, Table 1). The TIA cohort had a slightly higher level of education (>12 years, 39.0% vs. 37.4%) and a higher proportion in the highest income tertile (39.3% vs. 32.4%) compared with controls. However, the proportion of non-employed individuals was also higher among those with TIA (20.2% vs. 17.0%).

**Table 1:** Characteristics of individuals with transient ischemic attack and age- and sex-matched controls.

Characteristics	TIA cohort n=347 (20%)	Control cohort n=1388 (80%)
Age, median (range) years	59 (26-66)	59 (26-66)
Sex, n (%)		
Men	206 (59.4)	824 (59.4)
Women	141 (40.6)	564 (40.6)
Sick leave, n (%)		
30 days before	28 (8.1)	54 (3.9)
0 days (index date)	64 (18.4)	55 (4.0)
14 days after	124 (35.7)	47 (3.4)
2 months after	80 (23.1)	49 (3.5)
1 year after	40 (11.5)	53 (3.8)
Sick leave, mean (SD) days		
1 year before index date	26 (78)	14 (55)
1 year after index date	65 (114)	15 (62)
Disability pension, n (%)		
Before index date	51 (14.7)	156 (11.2)
1 year after index date	1 (0.3)	5 (0.4)
Country of birth, n (%)		
Sweden	263 (75.8)	1136 (81.8)
Outside of Sweden	84 (24.2)	252 (18.2)
Education, n (%) *		
< 10 years	52 (15.1)	217 (15.8)
10-12 years	158 (45.9)	643 (46.8)
> 12 years	134 (39.0)	514 (37.4)
Income, n (%) *		
Lowest tertile	115 (33.2)	453 (32.8)
Middle tertile	95 (27.5)	480 (34.8)
Highest tertile	136 (39.3)	448 (32.4)
Employment status, n (%) *		
Employed	276 (79.8)	1146 (83.0)
Non-employed	70 (20.2)	235 (17.0)
Comorbid conditions, n (%)		
Prior stroke	46 (13.3)	–
Atrial fibrillation	34 (9.8)	–
Hypertension	152 (43.8)	–
Diabetes	44 (12.7)	–
Smoking, n (%) *		
Yes	62 (19.4)	–
No	257 (80.6)	–
Prior physical activity, n (%) *		
Inactive	104 (36.5)	–
Physically active	181 (63.5)	–

% are based on available data. * Missing data: education (TIA: n = 3; controls: n = 14), income (TIA: n = 1; controls: n = 7), employment status (TIA: n = 1; controls: n = 7), smoking (TIA: n = 28), pre-stroke physical activity (TIA: n = 63).

There was a moderate correlation between the number of days on sick leave before and after TIA (ρ = 0.56, p < 0.001). The mean number of days on sick leave increased by 39 days during the year following TIA compared with the preceding year (95% CI 28–50 days, p < 0.001). Individuals with TIA also had more sick leave days compared to controls during the year before the event (mean 26 [SD 78] vs. 14 [SD 55], p < 0.001). The median number of sick-leave days was 0 (IQR 0–1) during the year before TIA and 0 (IQR 0–70) during the year after TIA, compared with 0 (IQR 0–0) during both periods in the control cohort.

At 30 days before the index date, 28 (8.1%) individuals in the TIA cohort and 54 (3.9%) of controls had ongoing sick leave. The proportion increased to 64 (18.4%) in the TIA cohort at the date of diagnosis and peaked 14 days after the index event, with 124 (35.7%) of individuals with TIA compared with 47 (3.4%) of controls on sick leave. By 2 months, the proportion of those with TIA on sick leave had declined to 80 (23.1%), and at 1 year, 40 (11.5%) remained on benefit, compared with 49 (3.5%) and 53 (3.8%) of controls, respectively. Disability pension was obtained by 14.7% of individuals in the TIA cohort and 11.2% of controls before the index date, and few new cases were approved one year after (1 [0.3%] of individuals with TIA, and 5 [0.4%] of controls).

The number of individuals with ongoing sick leave in the TIA cohort and matched control cohort, stratified by underlying diagnosis category and time point are presented in Fig 1. The most common registered diagnoses for sick leave 14 days after the event among individuals with TIA was a TIA diagnosis (53 of 124 [42.7%]), followed by mental health related diagnoses (16 [12.9%]), and stroke (15 [12.1%]). After 2 months, TIA was still the most common diagnosis for sick leave (24 of 80 [30.0%]), followed by mental health (15 [18.8%]), and stroke (15 [18.8%]). After 1 year however, only 4 of 40 (10.0%) individuals in the TIA cohort who were on sick leave had TIA as the underlying diagnosis, whereas 9 (22.5%) had stroke and 14 (35.0%) had a mental health diagnosis. In the control cohort, the sick leave prevalence remained stable over time, with mental health–related diagnoses consistently being the most common underlying cause (Fig 1).

**Fig 1:**
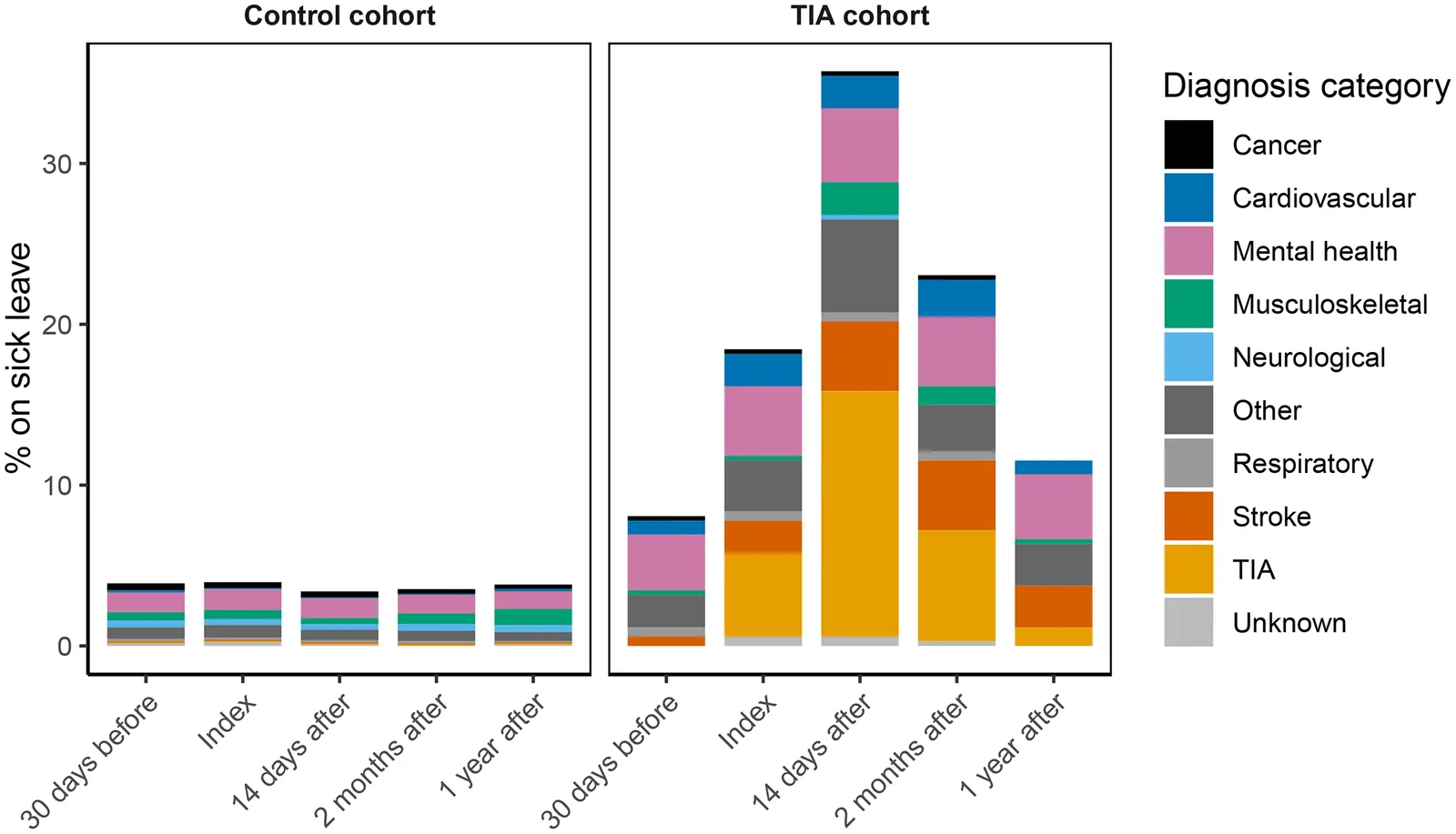
Number of individuals with ongoing sick leave in the TIA cohort and matched general population control cohort, stratified by registered diagnosis and time point. ICD-10 codes were classified into TIA (G45), Stroke (I61, I63, I64), Cardiovascular (other I codes), Neurological (other G codes), Musculoskeletal (M), Respiratory (J), Mental health (F), Cancer (C), Other diagnosis (all remaining codes), and Unknown diagnosis (no ICD-10 code reported). Time points represent 30 days before index, at index, 14 days after, 2 months after, and 1 year after the index event. Individuals with TIA (cases) and their age- and sex-matched controls are displayed separately.

In multivariable binary logistic regression analysis, higher income and active employment were associated with increased odds of ongoing sick leave 14 days after TIA (Table 2). Compared with individuals in the lowest income tertile, those in the middle (adjusted OR 2.54, 95% CI 1.22–5.29, p = 0.013) and highest tertiles (adjusted OR 2.11, 95% CI 1.03–4.34, p = 0.041) had greater odds of being on sick leave. Employed individuals also had higher odds of sick leave 14 days after TIA than non-employed individuals (adjusted OR 3.11, 95% CI 1.23–7.88, p = 0.016). Education and the other included variables were not significantly associated with sick leave.

**Table 2:** Associations between characteristics and sick leave 14 days after transient ischemic attack.

	No (%) /Median (range)	Univariable model		Multivariable model	
Variables		Odds ratio (95% CI)	P-value	Odds ratio (95% CI)	P-value
Age	58 (26-66)	1.02 (0.99-1.06)	0.166	1.03 (0.99-1.07)	0.081
Female sex	116 (39.3)	1.30 (0.81-2.09)	0.285	1.42 (0.87-2.31)	0.166
Born in Sweden	227 (76.9)	1.06 (0.61-1.85)	0.834	–	–
Income					
Lowest tertile	73 (24.7)	Reference	–	Reference	–
Middle tertile	87 (29.5)	3.22 (1.62-6.40)	<0.001	2.54 (1.22-5.29)	0.013
Highest tertile	134 (45.4)	2.37 (1.24-4.50)	0.009	2.11 (1.03-4.34)	0.041
Education					
< 10 years	37 (12.6)	Reference	–	Reference	–
10-12 years	131 (44.7)	2.35 (1.05-5.25)	0.037	1.79 (0.84-3.83)	0.134
> 12 years	125 (42.7)	1.52 (0.67-3.42)	0.313	1.11 (0.50-2.46)	0.805
Employment	117 (40.1)	3.60 (1.45-8.97)	0.006	3.11 (1.23-7.88)	0.016
Prior stroke	31 (10.5)	0.97 (0.45-2.09)	0.941	–	–
Atrial fibrillation	25 (8.5)	0.46 (0.18-1.19)	0.108	1.38 (0.61-3.14)	0.442
Hypertension	125 (43.4)	1.04 (0.65-1.67)	0.872	–	–
Diabetes	31 (10.5)	1.13 (0.53-2.40)	0.753	–	–
Smoking	46 (17.0)	1.01 (0.53-1.94)	0.971	–	–
Being physically active	161 (65.7)	1.16 (0.67-1.99)	0.598	–	–

Associations were calculated using univariable and multivariable binary logistic regression. The outcome was ongoing sick leave 14 days after a transient ischemic attack (n = 116). The multivariable analysis included 292 individuals with TIA, excluding individuals with disability pension (n = 51), and cases with missing data (n = 4). Nagelkerke R Square = 0.15. Hosmer and Lemeshow Test Chi-square = 7.48, p = 0.486.

## Discussion

In this longitudinal, register-based cohort study, more than one-third (35.7%) of individuals with TIA had ongoing sick leave 14 days after the event, compared with only 3.4% of randomly sampled matched controls. Hence, a substantial proportion of working-age adults with TIA experience temporary work incapacity immediately after the event, despite TIA being defined by rapid symptom resolution. Although the proportion decreased over time, one in ten individuals with TIA remained on sick leave one year later, suggesting lingering functional or psychological difficulties that may hinder full return to work.

The rapid peak and subsequent decline in sick leave suggest that most individuals experience only a brief period of work incapacity following a TIA. However, the persistence of sick leave in a subset of individuals indicates that some may have lasting sequelae affecting their ability to return to work. Similarly, a recent Danish register-based study reported that approximately one in five with TIA received sick leave benefits 5–12 weeks after the event and that nearly one in six of older individuals with TIA (aged 65–79) required homecare at three months.^7^ In comparison, nearly half of those with ischemic stroke were on sick leave during the same period. Their results, together with the current study, indicate that although return to work is faster after TIA compared to stroke, sick leave remains considerably common among individuals with TIA compared with the general population.

Mental health–related diagnoses were common causes of sick leave after TIA, aligning with prior studies reporting residual cognitive, emotional, or physical symptoms after TIA [11,12]. Although mental health–related diagnoses is a common cause of sick leave in the overall Swedish population [13], as well as among the randomly sampled control population in this study, the observed increase in such diagnoses at later time points after TIA highlights a potential need for enhanced psychological and psychosocial support in this group.

Higher income was associated with increased odds of sick leave 14 days after TIA. Normal population studies have reported an over-representation of low-income workers among those with high levels of sick leave in the Nordic countries [14]. However, this finding should not be interpreted as indicating greater post-TIA morbidity among individuals with higher income. Because the outcome reflects registered sickness benefit, the observed associations may partly reflect labour-market attachment, benefit eligibility, occupational demands, awareness of entitlements, and other socioeconomic differences in access to the benefit system. Individuals with higher income may also have jobs requiring greater cognitive workload, making even subtle deficits more limiting in their occupational context. Supporting the notion that cognitive deficits may affect work capacity, previous studies have reported that executive function can decline during the first six months after TIA [5^].^ In addition, quantitative job demands have been strongly linked to work-related fatigue after stroke [15]. Employment was also associated with sick leave, further suggesting that socioeconomic and occupational factors influence work absence after TIA.

The finding that TIA itself was the dominant diagnosis for sick leave shortly after the event supports the clinical relevance of the TIA diagnosis in the context of work incapacity. Individuals with TIA had more days on sick leave than their controls during the year before the event, indicating preexisting vulnerability or underlying morbidity. Prior research suggests that the impaired health in individuals diagnosed with TIA mostly reflect preexisting morbidity, and that TIA events are generally not associated with worsening of overall health [16]. Although hospitalization following TIA aims to prevent development of stroke, secondary prevention may still be unsuccessful in some individuals [17]. However, this should not necessarily be interpreted as evidence that the TIA itself directly caused the work incapacity, as the recent TIA diagnosis may have been used even when sick leave was related to cognitive, emotional, or other residual symptoms. One year after the event, stroke diagnoses were more common than TIA among those still on sick leave, suggesting either initial diagnostic uncertainty or the development of a subsequent stroke.

From a rehabilitation perspective, our findings suggest that the consequences of TIA extend beyond short-term neurological symptoms. Systematic assessment of work ability, cognitive function, and emotional well-being should be considered part of TIA management. Early identification of individuals at risk of prolonged sick leave may enable clinicians to implement targeted interventions and guide patients toward structured return-to-work programs, occupational therapy, and appropriate psychological support.

### Strengths and limitations

Strengths of this study include the use of high-quality, nationwide registers with near-complete coverage, minimizing recall bias and loss to follow-up [9,18]. The inclusion of a large, age- and sex-matched control group from the general population allows for comparisons and enhances external validity.

However, several limitations should be noted. First, the definition of TIA was based on clinical diagnosis and may include diagnostic uncertainty, as some cases may represent minor strokes or other transient neurological events. In addition, brain MRI is not always performed during the acute stage of care in Sweden, which may have led to undetected smaller infarctions and diagnostic misclassification. Second, although sick leave data are objective, they reflect physician-certified work incapacity rather than direct measures of health or function, and differences in health literacy may influence results. Third, the TIA cohort was drawn from a single urban region and three stroke units within one university hospital. Regional differences in population characteristics, sick-leave practices, labour-market conditions, and access to MRI may limit generalizability to other settings. Fourth, while the study controlled for key sociodemographic variables, unmeasured factors include imaging and clinical follow-up data, personal and work-related factors as well as some comorbid conditions. Information on comorbidities and health-related characteristics, including prior stroke, atrial fibrillation, hypertension, diabetes, smoking, and physical activity, was obtained from stroke-specific registers and was therefore available in the TIA cohort. Consequently, we could not compare these characteristics between individuals with TIA and matched controls. Another limitation related to the control cohort is that the index date was an assigned reference date rather than a clinical event, preventing direct comparison of determinants of sickness benefit at 14 days between cohorts. Finally, the observational design precludes causal inference.

## Conclusions

Sick leave, indicating lasting work incapacity, was common after TIA, with 36% of working-age individuals receiving benefit two weeks after the event and 11.5% still on benefit at one year. Individuals with TIA also had more days on sick leave in the year preceding the event compared with matched population controls, suggesting preexisting vulnerability or underlying morbidity. Work incapacity after TIA cannot be attributed solely to the acute neurological episode but may reflect a broader pattern of reduced health and resilience in this group. Systematic assessment of functioning and targeted rehabilitation or return-to-work interventions can be considered to promote sustained occupational recovery after TIA.
